# Evolution, Ecology, and Zoonotic Transmission of Betacoronaviruses: A Review

**DOI:** 10.3389/fvets.2021.644414

**Published:** 2021-05-20

**Authors:** Herbert F. Jelinek, Mira Mousa, Eman Alefishat, Wael Osman, Ian Spence, Dengpan Bu, Samuel F. Feng, Jason Byrd, Paola A. Magni, Shafi Sahibzada, Guan K. Tay, Habiba S. Alsafar

**Affiliations:** ^1^Center for Biotechnology, Khalifa University of Science and Technology, Abu Dhabi, United Arab Emirates; ^2^Department of Biomedical Engineering, College of Engineering, Khalifa University of Science and Technology, Abu Dhabi, United Arab Emirates; ^3^Center of Heath Engineering Innovation, Khalifa University of Science and Technology, Abu Dhabi, United Arab Emirates; ^4^Nuffield Department of Women's and Reproduction Health, Oxford University, Oxford, United Kingdom; ^5^Department of Pharmacology, College of Medicine and Health Sciences, Khalifa University of Science and Technology, Abu Dhabi, United Arab Emirates; ^6^Department of Biopharmaceutics and Clinical Pharmacy, School of Pharmacy, The University of Jordan, Amman, Jordan; ^7^Department of Chemistry, College of Arts and Sciences, Khalifa University of Science and Technology, Abu Dhabi, United Arab Emirates; ^8^Discipline of Pharmacology, University of Sydney, Sydney, NSW, Australia; ^9^State Key Laboratory of Animal Nutrition, Institute of Animal Science, Chinese Academy of Agricultural Science, Beijing, China; ^10^Department of Mathematics, Khalifa University of Science and Technology, Abu Dhabi, United Arab Emirates; ^11^Department of Pathology, Immunology and Laboratory Medicine, University of Florida, Gainesville, FL, United States; ^12^Discipline of Medical, Molecular and Forensic Sciences, Murdoch University, Murdoch, WA, Australia; ^13^Murdoch University Singapore, King's Centre, Singapore, Singapore; ^14^Antimicrobial Resistance and Infectious Diseases Laboratory, College of Science, Health, Engineering and Education, Murdoch University, Murdoch, WA, Australia; ^15^Division of Psychiatry, Faculty of Health and Medical Sciences, The University of Western Australia, Crawley, WA, Australia; ^16^School of Medical and Health Sciences, Edith Cowan University, Joondalup, WA, Australia; ^17^Department of Genetics and Molecular Biology, College of Medicine and Health Sciences, Khalifa University of Science and Technology, Abu Dhabi, United Arab Emirates

**Keywords:** zoonoses, coronavirus, SARS-CoV-2, zoonotic transmission, ecology, evolution, reservoir species

## Abstract

Coronavirus infections have been a part of the animal kingdom for millennia. The difference emerging in the twenty-first century is that a greater number of novel coronaviruses are being discovered primarily due to more advanced technology and that a greater number can be transmitted to humans, either directly or *via* an intermediate host. This has a range of effects from annual infections that are mild to full-blown pandemics. This review compares the zoonotic potential and relationship between MERS, SARS-CoV, and SARS-CoV-2. The role of bats as possible host species and possible intermediate hosts including pangolins, civets, mink, birds, and other mammals are discussed with reference to mutations of the viral genome affecting zoonosis. Ecological, social, cultural, and environmental factors that may play a role in zoonotic transmission are considered with reference to SARS-CoV, MERS, and SARS-CoV-2 and possible future zoonotic events.

## Introduction

**Figure 1:**
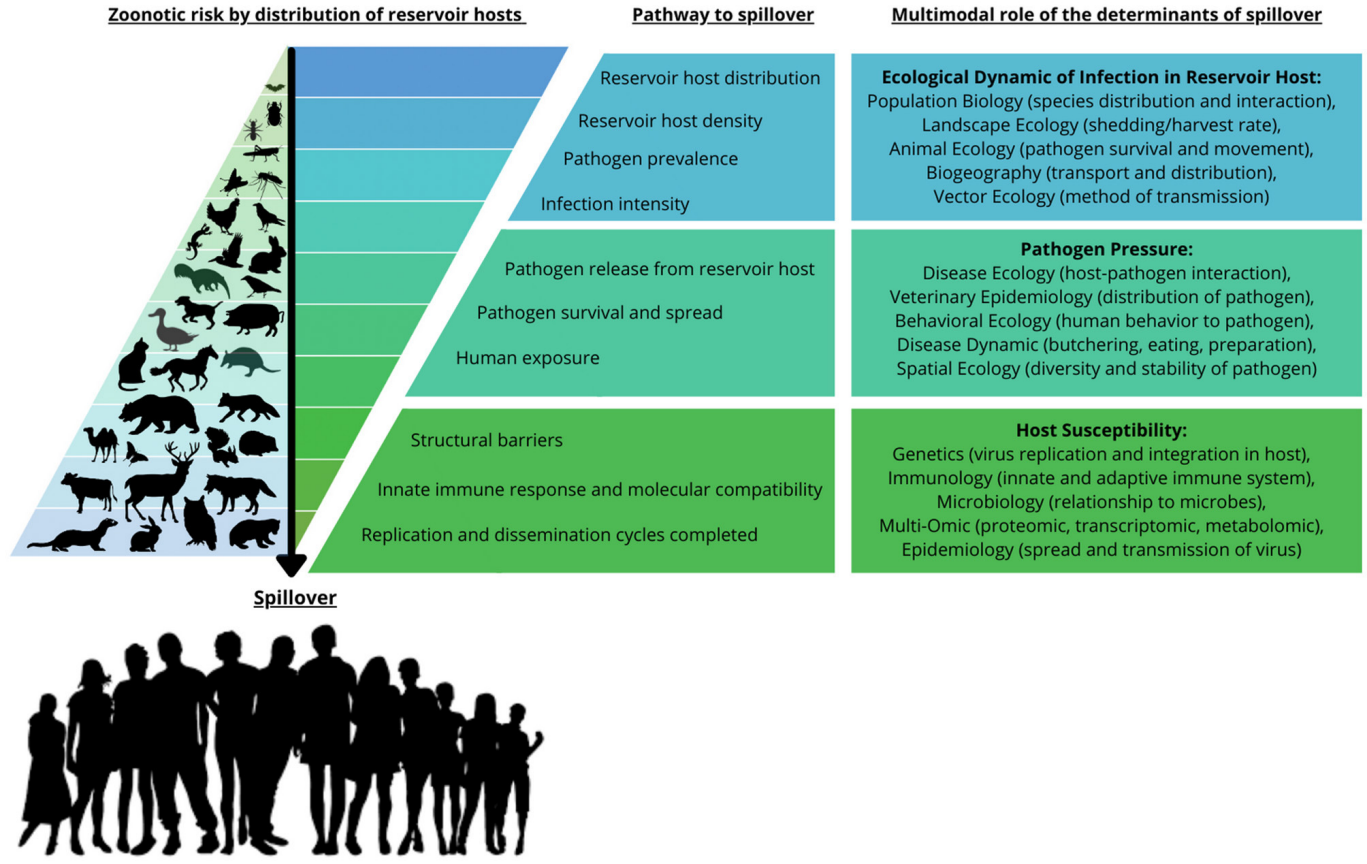
Zoonotic risk distribution, pathway to spillover, and the multimodal role of the determinants of spillover. The zoonotic risk is demonstrated by the accumulated distribution of reservoir hosts and vectors that play a role in the pathway to spillover. The risk of spillover is determined by a series of processes from the ecological dynamics of reservoir host distribution and density, to the susceptibility, replication and dissemination of the biological factors in the recipient host. This is also reflected in the multimodal role of the determinants of spillover, demonstrating the disciplines that are being used to study zoonotic transmission and the determinants of spillover. This figure was adapted from Figure 1 in Plowright et al. (2). Copyright permission was obtained with license number 55218980848529.

The emergence of the Severe Acute Respiratory Syndrome Coronavirus 2 (SARS-CoV-2), the virus responsible for the COVID-19 pandemic, has focused attention on the phenomenon of zoonosis. Zoonoses, as defined by the World Health Organization (WHO), are diseases and infections which are naturally transmitted between vertebrate animals and humans. The challenge with emerging zoonoses, such as SARS-CoV-2, is to establish the origin and mechanism(s) of transmission of the new disease. Viral mutation and recombination, viral host physiology and immune response, ecogeography, and human factors including ACE2 receptor structure and immune function have all been proposed as possible mechanisms (1, 2) (Figure 1).

Viruses account for ~25–44% of all emerging infectious diseases and present an increased risk of zoonotic transmission (3–5). Zoonotic viral transmission has been well established in the links between human immunodeficiency virus-1 (HIV-1) and simian immunodeficiency viruses (SIVs) from chimpanzees and gorillas in West Central Africa, and HIV-2 and SIVs from sooty mangabeys (6). Zoonotic virus to human transmission and the number of viral-based infections has been steadily increasing, prompting a worldwide investigation of potential zoonotic pathogens, not only to understand current zoonoses but, more importantly, to also identify potential future transmissions of viral pathogens between animals and humans (4, 5). The emergence of SARS-CoV, Middle East Respiratory Syndrome (MERS-CoV), and SARS-CoV-2, which are all highly pathogenic, is the end-point in a sequence of events involving viral evolution and ecology (7). Mutation, recombination, genetic drift, and other evolutionary mechanisms, combined with ecological pressures and opportunities, allow the viruses to cross several species barriers and overcome immune responses in the new hosts. However, the origin and the evolutionary changes of the SARS-CoV-2 virus in humans are currently unknown.

Evidence from other viral zoonoses suggests an origin in bats *via* possibly an intermediate host (8, 9). This review will examine the origin of SARS-CoV-2 in the context of possible zoonotic transmission, evolution, ecological factors, and the role of reservoir species in other coronaviruses (10).

### Coronavirus Characteristics

Coronaviruses are members of the family Coronaviridae, order Nidovirales. The Coronaviridae family is further subdivided into the Torovirinae and Coronavirinae subfamilies. Coronaviridae have a well-conserved genomic organization and a substantial number of nonstructural genes, as well as unique enzymatic activities (3, 11). The sub-family of Coronavirinae contains four genera: alphacoronavirus (α-CoV) and betacoronavirus (β-CoV), which infect mammals, and gammacoronavirus (γ-CoV) and deltacoronavirus (δ-CoV), which principally infect birds (12, 13). Coronaviruses (CoVs) are large enveloped positive-sense single-stranded (+)ssRNA viruses. Genomes of CoVs range from 25 to 32 kilobases (kb), and reproduction of this genome involves a unique transcription mechanism (14). CoV genomes contain five open reading frames (ORFs) encoding for the replicase protein 1a/1b nucleocapsid (N), spike (S), membrane (M), and envelope (E) proteins essential for the replication of the virus. CoVs also contain a variable number of ORFs coding for accessory proteins, not essential for replication but with possible roles in pathogenesis (15, 16).

SARS-CoV-2 belongs to the genus betacoronavirus (β-CoV). GenBank sequences of β-CoVs from the first patients in the Chinese city of Wuhan, who visited the seafood market, indicated that bat-SL-CoVZC45 and bat-SL-CoVZXC21 collected in 2018 in Zhoushan, eastern China, have the highest sequence identities (~90% similarity) with SARS-CoV-2. When gene regions were analyzed individually, the S gene (Spike protein) had the lowest sequence identity (75%), and the E gene the highest (98.7%). Significantly, the SARS-CoV-2 strains in the initial Chinese study of nine patients had a sequence identity of only 79% with SARS-CoV and ~50% with MERS-CoV (17). These findings suggest that SARS-CoV-2 is a distinct β-CoV within the Coronaviridae family (18). However, SARS-CoV-2 does share a similar ACE2-binding domain with only a few amino acid differences from SARS-CoV (19, 20). Later sequencing of SARS-CoV-2 from Taiwan samples indicated further differences from SARS-CoV, especially in the ORF8 loci (21).

Within the Coronavirinae subfamily, different host species are involved with different transmission profiles, and not all have demonstrated zoonotic transmission to humans at this time. Of the seven CoVs which have transferred to humans, two (HCoV-229E and HCoV-NL63) are α-CoVs (22, 23). HCoV-OC43, HCoV-HKU1 (24, 25), SARS-CoV, MERS-CoV (26–29), and SARS-CoV-2 are β-CoVs (30, 31). Infections with HCoV-229E, HCoV-OC43, HCoV-HKU1, and HCoV-NL63 are usually self-limiting with mild symptoms (5, 25, 32–36). Although HCoV-OC43 infection is usually mild, more severe pathologies, including fatal pulmonary infections and lethal encephalitis, have been reported (37). SARS-CoV-2 does not have any close sequence similarity to HCoV-229E, HCoV-OC43, HCoV-HKU1, or HCoV-NL63 but is antigenically related to SARS-CoV (38, 39).

Entry to host cells by CoVs, including SARS-CoV, MERS, and SARS-CoV-2, is mediated *via* the spike protein (40). The S1 subunit of this protein binds to a receptor on the host cell surface, and the S2 subunit fuses the viral and host membranes (41). An important property of CoVs is the diversity in receptors recognized by the S1 protein in different viruses. These include zinc peptidases angiotensin-converting enzyme 2 (ACE2) (SARS-CoV; SARS-CoV-2; HCoV-NL63), aminopeptidase N (APN) (HCoV-229E), the serine peptidase dipeptidyl peptidase 4 (DPP4) (MERS-CoV), 9-O-acetylated sialic acid (9-O-Ac-Sia) (HCoV-HKU1; HCoV-OC43), a cell adhesion molecule carcinoembryonic antigen-related cell adhesion molecule 1 (CEACAM1) as is the case with mouse hepatitis virus (MHV), and an angiotensin II receptor type 2 (AGTR2) possibly with SARS-CoV-2 (42–45). Other species use sugars as receptors or co-receptors (41). Apart from the ACE2 receptor-binding site for virus entry, TMPRSS2 is another key protein required for viral entry into human tissue (46–50).

Some spike proteins may also have different access routes to cells of possible host species. PDF2180-CoV, a MERS-like virus found in a Ugandan bat, does not use the DPP4 receptor for cell entry but requires proteolytic cleavage using possibly gastrointestinal tract (GIT) released trypsin; it, then, may enter *via* the digestive tract rather than *via* the respiratory system (51). The MERS DPP4-binding site has also been shown to have natural polymorphisms (K267E, K267N, A291P, and Δ346-348) that reduce cell entry capacity of the virus and two polymorphisms (K267E and A291P) that reduce viral replication (52). SARS-CoV-2, HCoV-OC43, MERS-CoV, and MHV-A59 are characterized by a furin-like cleavage site that may be important in virus cell entry since furin is found in large amounts in the lung (53, 54). In addition, potential routes of transmission of SARS-CoV-2, including entry *via* oral, sexual, and ocular pathways, cannot be discounted due to the wide spread of the ACE2 receptor in different tissues and organs (55, 56). MERS-CoV has already been shown to have multiple transmission and infection options by entering cells within endosomes or at the plasma membrane, depending on the location of the host proteases that cleave the viral spike protein to allow membrane fusion (57).

#### Coronavirus Evolution

Evolution-associated genotypic and phenotypic changes are a function of complex regulatory cellular, organismic, environmental, and geographical interactions (58). Coronaviruses and other zoonotic viruses are genetically heterogeneous while in prehuman hosts, such as bats, but are often poorly adapted for zoonotic transmission from their initial reservoir pool. This is due to physiological differences between host species, and hence requires significant evolutionary adaptation, which is often a co-evolutionary process (59, 60). Evolutionary changes associated with mutations and recombination, as well as genetic drift or pleiotropy, play an essential role in the adaptation of viruses to new hosts. These changes may keep viruses not only in the host population but also determine their virulence as seen in the adaptive evolution of the S glycoprotein of bovine coronavirus and human coronavirus (HCoV) OC43 using phylogenetic and phylodynamic analysis (61–63).

For successful transmission to a new species, viruses must adapt to cell surface receptors to bind successfully to the host receptor, escape host immune surveillance and response, and ensure further transmission by the host (64). Successful viral cross-species transmission and infection depend on rapid evolution accomplished by high mutation rates, recombination, and assortment and how closely related possible host species are (17, 65–68). Mutations that favor more efficient host entry, optimize virus replication, and decrease susceptibility to host immune responses, will increase virulence and maximize transmission potential either within the host species or between hosts (69–71). Findings of previous cross-species transmission has identified the importance of tracking viral mutations in the new host, in SARS-CoV-2 mutations to the receptor-binding domain changes virus virulence, and either natural immune response or vaccine efficacy (72).

Modification of the CoV genome and associated structural and non-structural proteins contribute to successful zoonoses (73–76). These genomic changes are further enhanced by geographical spread (77). Mutation rates in viruses are generally much higher (by a factor of ~10^6^) (78) than those of their hosts but vary for different viruses by several orders of magnitude (79). Estimated mutation rates in CoVs are moderately higher than others in the ssRNA virus category (78). In addition, SARS-CoV and SARS-CoV-2 may have evolved rapidly due to different parts of the genome mutating at different rates leading to a possible increase in phenotypic diversity (80, 81). Mutations may also lead to viral resistance to host immune reactions and resistance to antiviral drugs (82, 83). Forty-seven SNPs (point mutations) that may affect virulence have been identified in samples of SARS-CoV-2 from 12 countries from which complete genome sequencing data were available (84). Mutations in the sequence of the spike protein have led to the great versatility of viral receptor binding strategies, which characterizes the SARS-CoV and SARS-CoV-2 spike proteins. This versatility may underlie their high, yet different, affinities to the human ACE2 receptor compared with that of the spike protein in Bat-CoV and affect virulence and transmission (82, 85–87). However, mutations also lead to disparity in fatality rates in different countries following SARS-CoV-2 infection, which has been linked to the D614G substitution in the SARS-CoV-2 spike (S) protein suggesting changes in pathogenicity and transmission associated with specific mutations (21, 88–90).

Major drivers of CoV evolution also include mutation rate, viral mutation capacity, and recombination, influenced by environmental changes, as well as surface glycoprotein plasticity often promoted by host-parasite interaction without reducing virulence (91, 92). Polymorphisms of specific genes, however, do not only change the possible phenotypic expression of the gene but may also influence other genes and their expression. Genetic polymorphisms that affect host phenotype upon infection have been reported for HIV-1, hepatitis C, dengue, influenza A virus infections, and other types of parasites (93, 94). These studies, as well as plant studies, suggest that viruses may have both virulence and avirulence genes that map to susceptibility and resistance genes in the host (94). Improved immunity, for instance, has been found for the cellular co-receptor CCR5, which prevents HIV-1 from entering the cell, leading to resistance against HIV-1 in some individuals (95). Similarly, the human leukocyte antigen (HLA) polymorphisms may be associated with resistance or susceptibility to viral infection (96). SARS-CoV and SARS-CoV-2 both enter cells *via* the ACE2 receptor and access to or ability to bind to ACE2 may be associated with zoonotic transmission potential to humans (60, 97). This is particularly interesting as the ACE2 gene is located on the X-chromosome; hence, females may potentially be heterozygous with different susceptibility to males who are hemizygous (98). ACE1 is a similar receptor with a similar function; it is characterized by an insertion (I)/deletion (D) polymorphism where the D allele is associated with reduced expression of ACE2 and therefore may confer some resistance to SARS-type viruses by changes in the ACE1/ACE2 balance (98–101). Similarly, two mutations affecting the non-structural protein 6 (NSP6) and the ORF 10 may lead to a lower virulence and pathogenicity of SARS-CoV-2 due to a decrease in protein stability with similar mutations within the receptor-binding domain (RBD) required for retaining viral strength and virulence (102, 103). A recent study also identified a novel SARS-CoV-2 mutation in the RBD furin-associated cleavage site that may lead to a decrease in virulence and transmission and is associated with the sequence of ZJ01 (BataCov/Zhejiang/ZJ01/2019) in a mild case of COVID-19 reported in China (104). Novel CoVs have been identified in bats in Vietnam (105) and Mexico, where 13 distinct CoVs (nine α-CoVs; four β-CoVs) were identified, 12 of which were novel (106). These observations highlighted the fact that bats may not only carry multiple viruses but harbor distinct CoVs across different geographical regions. Sequence homology between the novel and known viruses underscores the importance of identifying the viral reservoirs in different bat families (42).

The evolution of the virus does not occur in isolation but is accompanied by alterations in the host. Host evolution and ecology associated with genetic mutations and recombination events provide the necessary host factors that need to occur in parallel with viral evolution (107–109). For effective zoonoses and the ability to establish in a new host, several factors need to come together, including the emergence of a founder virus that possesses a modification and allows more efficient infection/transmission in the new host, as well as host alterations. For example, bats have to have a specific immune response that allows them to survive following viral infection, and not affect virus survival, viral load, or viral spill over. In bats, this may be associated with the type I interferon and interferon-associated antiviral activity (5, 110, 111). These evolutionary adaptations are linked to damping of cytoplasmic DNA through the loss of PYHIN genes and a regulatory site mutation in STING, which leads to changes in the inflammatory response in association with a change in TNFα, IL-10, IL-1β, and IL-18, and IFN-α levels (112, 113). Recent studies have also shown that frequency and synchronization of the reproduction cycle of bats can influence the prevalence and persistence of viruses. The Egyptian fruit bat, *Rousettus aegyptiacus* E. Geoffroy, tested in Africa indicated two peak periods of horizontal transmission throughout the year and coincidental increase in human infections of Marburg virus, which is carried by these bats (114).

Persistence in the new host requires further genetic modifications. Mechanisms including repressor-operon function in gene action, the role of viruses incorporated as extrachromosomal elements, recombination, reassortment, codon bias, hypermutations, and epigenetics in host evolution all play a role in either resistance or susceptibility in viral infections including zoonoses (115, 116). In particular, epigenetic modifications by viruses, including CoVs, such as histone modifications, may influence host viral susceptibility (117). In this regard, following the zoonotic introduction of HCoV-OC43 to humans, the hemagglutinin-esterase protein required for binding to the 9-O-acetylated sialic acid as receptor was selected against contributing to the persistence of the virus in the human population (118). In addition, rapid recombination and mutations have led to a diversification of HCoV-OC43, with seven genotypes identified. In contrast, bovine coronaviruses (BoCoV) exhibit relative genetic stability that may be similar to porcine hemagglutinating encephalomyelitis virus (PHEV) (119–126).

In the case of SARS-CoV-2, the large ssRNA genome provides the virus with a multitude of pathways for adaptation-associated base substitutions and deletions and contributes to altering host gene expression and counteracting the host immune responses (127, 128). The previous pandemic associated with SARS-CoV did not identify a direct evolutionary pathway toward zoonotic transmission as no evidence has been found for mutations from a known coronavirus, nor does it seem to be a recombination or mutation of any known CoVs suggesting that SARS-CoV possibly spilled over into an intermediary, unidentified animal, bird, or reptile host prior to infecting humans (129, 130). A similar lack of identification of an intermediary host for SARS-CoV-2 is the case.

## Ecological Factors And The Role Of Reservoir Species

Zoonoses depend on the relationship between the virus and a reservoir host or hosts. Both the ecology of the virus and the ecology of the host(s) play roles in creating the potential for zoonotic transmission. Viral ecology is concerned with the interactions between the virus and its host or hosts but broader ecological factors affecting the host(s) directly play a role in the ecology of viruses. Viral ecology is an important link in the process of zoonoses as ecological factors can facilitate or inhibit virus spillover (131, 132). Comparisons of viral sequence data with demographic and geographic sampling location data can assist in our understanding of current and potential zoonoses (133). Animal to human transmission opportunities have been increasing in line with ecological changes worldwide (134–139). Zoonotic transmission risk is higher where population growth has had the greatest effect on land-use, and biodiversity is high (77, 140, 141). A global heat map from 2017 indicated that China, the Indian subcontinent, and the Himalayan region are hot spots with an increased risk of future zoonotic events (77). Although viruses play a role in broader environmental ecology, this discussion will focus on examining interactions of CoVs with identified host species and the types of transmission cycles that have evolved with respect to zoonoses.

Recent studies have indicated that viral diversity is reflective of the number of natural host species. Rodentia (rodents) and Chiroptera (bats) contain the most species among mammals (142). With over 1,400 bat species, bats are one of the most common mammals on the planet and have been identified as being likely associated with zoonosis (143). Bats form an important part of many ecosystems by regulating possible crop pest outbreaks, seed dispersal, and pollination, as well as soil fertilization, and as a food source are a major intermediate host of coronavirus species (144). The islands of the West Indian Ocean, which are home to 50 of the 1,411 known bat species provide a suitable environment for the co-evolution of hosts and viruses (145). Approximately 9% of the bat species in the West Indies tested positive for coronavirus of which nearly half were members of the α- and β-CoVs subfamilies. Evidence of co-evolution was shown with limited species switching, possibly due to habitat separation. The potential for spillover in this environment, however, remains to be investigated (144).

Viral transmission to humans may occur through the consumption of reservoir species for food or medicinal use of animal products from reservoir species as well as other commercial enterprises. Transmission of MERS-CoV, where the only identified host species are Dromedary camels, has been attributed to consumption of unpasteurized camel milk, raw meat, or medicinal use of camel urine, as well as proximity to the animals (146). Ecological factors thus facilitate the persistence and the frequent transmission of a virus in the case of MERS and dromedary camels (147). Consumption of wild-caught animals may not be necessary for transmission to humans as simple proximity to the host species appears to be sufficient for transmission in the case of SARS-CoV from palm civets and mink (148, 149). Other potential sources of contact between humans and wild animals occur when these animals are used for entertainment, as is the case for performing monkeys in Indonesia or zoos (150, 151). Thus, behavioral and cultural factors, which alter the probability of interactions between humans and animals have increased the possibility of cross-species infections, which may lead to more zoonotic events (66, 131, 142, 152–154).

Dispersal and host spillover dynamics are a function of animal ecology. Bats, birds, and especially migrating birds, which are hosts of CoVs, provide an opportunity for dispersal and spillover across large distances, continents, and host species. Feeding habits in airborne animals are constrained by the aerodynamics of flight and associated energy requirements and location of food sources as well as geography. Bats may either be frugivorous or insectivorous, and the fecal contents of bats may be ingested by ground-foraging animals, leading to transmission of the virus (155). An interesting corollary is that flight also leads to greater energy expenditure and higher body temperatures in bats, which has required viruses to adapt and led to a greater diversity of zoonotic viruses in bats (156, 157). The adaptation as part of a co-evolution may then have led to better resistance to inflammatory reactions, as part of the innate immune response in spillover species such as humans (158).

Warm-blooded flying vertebrates appear to be ideal hosts for coronaviruses, bats for alpha and beta CoVs and birds for delta and gamma CoVs (159). Migratory birds are known to have led to transfection of the West Nile virus, tick-borne encephalitis virus, influenza A virus (IAV), Newcastle disease virus (NDV), and H5Nx avian influenza viruses to humans (160, 161).

Bat ecogeographical characteristics, including food choices, population dynamics, territorial range, migration patterns, a long-life span, virus immunity, and the human-led changes in habitat that brings bats in more contact with humans, lead to bats being an ideal candidate to spread viruses to humans. Viruses with a diverse host range and host plasticity are also more likely to amplify viral spillover by secondary human-to-human transmission (31, 162–164). Shifts in the migratory behavior of species can alter the risk of infection for wildlife, domestic animals, and humans. These shifts in migratory behavior occur in conjunction with changes in food availability due to natural ecological change, agriculture, or feed supplementation (165). An increased risk of infection may occur in smaller regional areas if animals become sedentary and long migrations do not cull infected hosts but reduce geographical infection spread. Climate change and deforestation have the potential to cause many such ecological changes in the immediate future with increased risk of zoonotic events. Habitat deterioration, which may have detrimental effects on the health of the reservoir species, is an important adjunct to viral spillover probability, leading to higher susceptibility to pathogens and increases in shedding of virus (166). The extent that habitat deterioration or human activity affects bat species health is quite specific and depends on the roost type and human activity with respect to the roost type (167).

## Reservoir Species

A factor permitting viruses to persist in one host and, possibly, to cross to another is the presence in different hosts of common receptors. For movement across species, host evolution and ecology associated with genetic mutations and recombination events are the necessary host factors that need to occur in parallel with viral evolution (107–109). The cross-species infection can take several forms, but a common model is the spillover of the virus from bats through undigested food. Bats mainly break down food to obtain the high-energy sugars and spit out the remaining fruit which is consumed by other species including insects, which are a potential intermediary transmission vector depending on the bat species (168). Direct human transmission may also occur through eating bats and the use of bat feces for medicinal purposes (66, 169).

Studies from China in the last 15 years have shown that bats are the natural reservoir of a range of CoVs, including SARS-CoV and SARS-like CoVs, found in *Rhinolophus macrotis* Blythe, *Rhinolophus ferrumequinum* Schreber, *Rhinolophus pearsoni* Horsfield, *Rhinolophus sinicus* Andersen, *Pipistrellus abramus* Temminck, *Pipistrellus pipistrellus* Schreber, *Tylonycteris pachypus* Temminck, *Myotis ricketti* Thomas, and *Scotophilus kuhlii* Leach (136, 170–173). However, despite the high numbers of bat species harboring diverse CoVs, any direct link between these viruses and their bat hosts with viruses isolated from human samples is lacking. Several possibilities exist including host shifts between closely related hosts and/or viruses found in host species. Preadaptation of a virus strain to overcome the immune response of a new host is a mechanism that allows spill-over. In addition, a previrulent virus entry into a new host can allow mutations in the new host that eventually lead to increased virulence. These mechanisms are further outlined here with respect to reported studies of coronaviruses. From phylogenetic association studies, results show that for instance there is no strict match between Rhinolophid bats and their association with CoVs, suggesting that interspecies transmission followed by establishment and long-term persistence in the new host species have occurred during the evolutionary history of the virus rather than the current virus genome establishing in the human population (31, 119, 120). Host shifts within Rhinolophid bats have been identified in all bats harboring two distinct lineages of CoVs, except for *R*. *macrotis* (119, 135). This indicates that there is a divergence in behavior and ecogeography of the close phylogenetic relationship between viruses harbored by *M. ricketti* and *S*. *kuhlii* and reflects the similar behavior and ecology of the *R. ferrumequinum, R. pearsoni*, and *R. sinicus* hosts. Novel bat species have also been recently reported harboring CoVs being Myotis (*Myotis pequinius* Thomas) and Fringed long-footed Myotis (*Myotis fimbriatus* Peters). In addition, the genotypes of lineage C, β-CoV in Fringed long-footed Myotis (*M. fimbriatus*), and common serotine (*Eptesicus serotinus* Schreber) in China have a nucleotide similarity of 85–88% with MERS-CoV (124). Since viruses have probably evolved more recently than their host bats, the apparent co-evolutionary patterns could be due to a host shift that would allow viruses to undergo preadaptation against the immune responses of the host. This mechanism may explain the movement of SARS-like CoVs between hosts (121, 174).

Interspecies spill-over and viral recombination may lead to eventual zoonotic events that can also be viewed from the receptor binding domain of the virus with the ACE2 receptor perspective. Recent data suggests that the RBD for ACE2 may be an ancestral trait of SARS-CoV-2 and not a result of a recent recombinant event. Thus, SARS-CoV-2 may have diverged from its ancestral bat sarbecovirus reservoir sometime between 1948 and 1982 and circulated in bats for some time (175). Intriguing is the proposition that RaTG13 CoV with a high nucleotide sequence identity with the Spike protein of SARS-CoV-2 may not be the historic ancestor, as suggested by some reports but that the RBD was acquired from the MP789 pangolin CoV by recombination (176).

### MERS-CoV

MERS-CoV, which causes respiratory-type illness but differing from that caused by SARS-related coronavirus (SARSr-CoV) was isolated in 2012 (27, 177). MERS-CoV replicates in cell lines from Rousettus, Rhinolophus, Pipistrellus, Myotis, and Carollia bats as well as pigs, substantiating that MERS-CoV uses a different receptor to ACE2 that is conserved in bats, pigs, and humans, demonstrating a continued strong zoonotic potential (178, 179). Phylogenetic characterization has placed MERS-CoV into the β-CoV group (180, 181). No animal reservoir for MERS-CoV has been identified. The closest relatives of MERS-CoV occur in a bat species *Neoromicia zuluensis* Roberts (182–184). It is closely related to the Tylonycteris bat coronavirus HKU4 and Pipistrellus bat coronavirus HKU5 (27, 159). The closest viral relative to MERS-CoV sequence identity with MERS-CoV EMC/2012 (MERS coronavirus Erasmus Medical Center/2012) isolated in the infected person in Saudi Arabia has been reported for *Taphozous perforatus* E. Geoffroy (Egyptian Tomb bats), suggesting a possible regional host reservoir (182, 185). Studies of the evolutionary origins of MERS-CoV link *Pipistrellus cf. hesperidus* (strain PREDICT/PDF-2180) from the bat family Vespertilionidae as a possible evolutionary ancestor for MERS-like CoV, indicating a 30% homology between the MERS-CoV S protein antigenic sites and HKU4, but a 70% homology with HKU5 bat-CoV. A 100% homology between MERS-CoV and both HKU4 and HKU5 was found for the E, M, and N protein antigenic sites (157, 186, 187). Cross-reactive antibodies to MERS-CoV have been detected in dromedary camels, which have been suggested as an intermediary host, in Oman and the Canary Islands, but no virus was detected in these animals (188, 189). Another possibility has been suggested by Zhang et al. that at least in Kenya, co-infection with MERS-CoV and a HKU8r-CoV in camels occurred, leading to the possibility of recombination events between a precursor virus of MERS-CoV and HKU8r-CoV (190). Apart from dromedary camels, other new-world camelids, including alpacas, llamas, and Bactrian camels, are also at risk of MERS-CoV infection. The DPP4 receptor in Bactrian camels has a 98.8% sequence identity with the dromedary camel DPP4 receptor (191).

The precise mode of transmission for MERS-CoV, therefore, remains uncertain due to differences in the viral genome reported from different geographic regions (110, 192–198), including differences between the original African MERS-CoV and the current Saudi Arabian MERS-CoV clade (29, 199–201). MERS-CoV differs from the closely related bat viruses in binding to the DPP4 receptor, also referred to as CD26 cellular receptor (202, 203) found in humans, dromedaries, and pigs (204–206). The potential of MERS-CoV to undergo rapid alterations in the surface charge of the spike protein enhances viral entry and demonstrated that MERS-CoV utilizes multiple paths to adapt to changes in the receptor sites of host species (207–209). A study of naturally occurring DPP4 protein variants indicated that four (K267E, K267N, A291P, and Δ346-348) reduced binding and cell entry of MERS-CoV and two polymorphisms (K267E and A291P) attenuated viral replication (52). Whether human DPP4 variants differ in geographical location or between ethnic groups remains to be investigated and may shed a better light on why MERS-CoV infection in the African continent is less than in the Middle East. An alternative explanation of the low zoonotic potential of MERS-CoV to humans may be related to the level of human DPP4 receptor expression (210). In contrast, viral escape mutations as an adaptive mechanism toward neutralizing antibodies in humans have been shown to most often lead to a reduction in MERS-CoV fitness and DPP4 binding (211). Similar mutations and recombination events are also characteristic of SARS-CoV and SARS-CoV-2 (1, 11, 30, 212). The mechanisms that lead to a balance between enhanced transmissibility associated with the rapid mutations observed in the Spike protein gene to DPP4 variants and adaptive mechanisms, for neutralizing host immune response that lead to a reduction in viral fitness, has yet to be elucidated (213).

### SARS-CoV

SARS-CoV, discovered in 2003 in association with patients being admitted to hospitals with severe acute respiratory syndrome obtains human cell entry through the ACE2 receptor (214). SARS-CoV shows a close relationship to murine hepatitis virus (MHV) based on phylogeny but MHV binds to the murine receptor CEACAM1 (215). The structure of the RBD of SARS that binds to ACE2 reveals possible sites of importance for crossing to humans (216–218). The pathway of zoonotic transmission however remains unknown despite intensive investigations and the discovery of closely related viruses that have the capacity to infect multiple hosts using the ACE2 receptor (219). Interspecies similarities have been reported with the RBDs of civet and humans differing only in four positions. Amino acids at these positions are responsible for the imbalanced salt bridges at the hydrophobic virus/receptor interface leading to a 1,000-fold difference in affinity of the respective spike protein for human ACE2 that creates the observed species barrier for zoonosis (220, 221). This makes the civet an unlikely candidate for intermediary host. Arguments for civets as an intermediary host therefore hinge on the rapid stepwise mutation rate of SARS-related CoV that contributed to SARS-CoV adapting to the human RBD within a short period (222, 223).

Any viral mutations must then be non-virulent to the reservoir or host species. Of interest is that civets showed clinical signs when infected with two strains of human SARS-CoV and suggesting a complex relationship between virus and civets as possible intermediate hosts (219). More recent research has identified two viruses, RsSHC014 and Rs3367, which are closely related to SARS-CoV in horseshoe bats (*R. sinicus)* from Yunnan, China that also have a close similarity in the RBD of the spike protein. One, Rs3367 demonstrated that it was equally able to infect HeLa cells expressing ACE2 from human, civet and Chinese horseshoe bats. Rs3367 has 99.9% genome identity with CoV-WIV-1 (224). This suggests that horseshoe bats may not only be a natural reservoir for SARS-CoVs and SARS-like CoVs but future virus mutations, may be able to directly cross to humans without spilling over into intermediate species such as palm civets (*Paguma Larvata* C.E.H. Smith) or raccoon dogs (*Nyctereutes procyonoides* Gray) (222, 225). Continued research by Ge and co-researchers have also published a newer WIV mutation, SARS-like CoV-WIV16 (SL-CoV-WIV16), which has an identical gene organization to SL-CoV-WIV1 but a slightly different organization to that of civet SARS-CoV and other bat SL-CoVs. SL-CoV-WIV16 has a 96% nucleotide sequence identity with human (SARS-CoV GZ0 human isolate from 2003 SARS outbreak) and civet SARS-CoVs compared with SL-CoV-WIV1 with 83% nucleotide sequence identity (226). The large genetic diversity of ACE2 among bats and humans and the rapid mutation rate seen in coronaviruses and the often close similarity of the virus RBD with human ACE2 receptors suggests possible future zoonotic events either directly from bats as the reservoir species or *via* intermediary species including cat (*Felis catus* Linnaeus), red fox (*Vulpes vulpes* Linnaeus), and the Chinese ferret badger (*Melogale moschata* Gray), all found in market places (12, 227–230).

### SARS-CoV-2

SARS-CoV-2 is a novel recombinant virus for which the ancestral host species or intermediate spillover species is as yet unidentified, although horseshoe bats (*R. sinicus*) across China, Europe, and Africa harbor SARS-CoVs (163, 231). Two SARS-CoV-2-related bat coronaviruses (BatCoV RmYN02 and BatCoV RaTG13) have been identified in horseshoe bats (*Rhinolophus malayanus* Bonhote and *Rhinolophus affinis* Horsfield). BatCoV RaTG13 sampled from *R. affinis* in Yunnan in 2013 displayed an approximate 95% similarity to SARS-CoV-2, suggesting that SARS-CoV-2 may have crossed from bats in this area of China (51). Several studies, including one investigating the polyprotein 1ab (pp1ab) of CoVs that is involved in replication and transcription of the viral genome and virus sequence similarity, has identified bat SARS-CoV RaTG13 as being the closest to SARS-CoV-2, rather than CoVZXC21 and CoVZXC45, which also show some similarity to SARS-CoV-2 (51, 81, 232, 233). RaTG13, which has the most closely related CoV sequence to SARS-CoV-2 (96.2%), still has a distinctive RBD which is less efficient in binding to ACE2 (45, 51) and phylogenetic tree and haplotype network analysis suggest historic divergence between SARS-CoV-2 and RaTG13 CoV between 40 and 70 years ago (176, 234).

The animal origin of SARS-CoV-2 is possibly quite diverse, with several SARSr-CoVs having been detected in horseshoe bats co-located in a single cave in China (224). Several sequential recombination events may then have led to changes in the coding sequences of the S-protein, ORF3, and ORF8 of SARSr-CoVs in these bat species prior to spillover to the civet as a possible intermediary host and the emergence of SARS-CoV variants including SARS-CoV and SARS-CoV-2 (45, 235). In the same cave, further SARSr-CoVs with different S protein sequences that were able to recognize ACE2 were identified that have the potential to spillover to humans, including SARS-Like CoV-WIV1 (SL-CoV-WIV1) and β-GX210 found in Cynopterus sphinx in Guangxi (224, 236–238). Human ACE2 is one of the most polymorphic genes, with 317 missense single-nucleotide variations (SNVs) identified that play an important role in susceptibility to SARS-CoV-2 due to changes in binding affinity of the virus to the receptor (230). The diversity of the ACE2 receptor may lead to a greater likelihood of possible infection with future SARS-CoV-2 variants that currently may not be as infectious in the majority of the population. SARS-CoV-2 mutations are identified in South Africa and UK that may be spreading faster and with different infectivity (239). India has also revealed dispersion of SARS-CoV-2 into at least three clades with mutations found in the spike, RdRP, and nucleocapsid coding genes (89). A further possibility is the T265I mutation in the USA which aids SARS-CoV-2 survival in the host cells and may play a role in virulence, transmissibility, or infectivity (240). Whether the viral spread and infectivity is solely due to the change in the spike protein structure or a combination of mutations in other viral proteins, as well as differences in the ACE2 receptors, is currently being investigated (241).

The CoVs from bats in Sichuan and Yunnan province in China also had sequence similarities with SARSr-CoV including β-YN2018A, β-YN2018B, β-YN2018C, β-YN2018D, and β-SC2018 and between 92 and 97% sequence identity with the human SARS-CoV SZ3 (238). These findings indicate the capacity of the coronavirus to undergo recombination between different parts of the genome in different hosts. Such analysis helps to elucidate the possible role of Malayan pangolins (*Meloidogyne javanica* Treub) as an intermediate host, between bats and humans (242, 243). CoV sequence similarities have identified a virus cluster, including 6 pangolin CoVs (MP789, PCoV-GX-P5L, P5E, P1E, P4L, and P2V) and two bat CoVs (RaTG13 and RmYN02 from *R. affinis* and *R. malayanus*, respectively). Pangolins, sampled from Guangdong province in China harbor the Pan-SL-CoV-GD with a genetic similarity of 91.2% to SARS-CoV-2 and others sampled in Guangxi province harbor the Pan-SL-CoV-GX with an 85.4% similarity to SARS-CoV-2 (244–247). Moderate similarity was reported on a genome level between SARS-CoV-2 and pangolin CoV samples using phylogenetic analysis, and the ACE2 receptor binding domain of current SARS-CoV-2 resembles most closely that of the pangolin CoVs from Guangdong. However, it is unlikely that SARS-CoV-2 has emerged directly from the virus isolates in pangolins but rather from an ancestral virus that may have had sequence similarity with RaTG13 CoV that may have obtained the S protein from the MP789 CoV (232, 233, 245, 248, 249).

Coronavirus can infect many animal species other than humans any of which could, conceivably act as an intermediate host species. Possibilities include snakes (Serpentes), hedgehogs (Erinaceidae), bats (Chiroptera), marmots (Marmota), turtles (*Chrysemys picta bellii* Gray, *Chelonia mydas* Linnaeus, and *Pelodiscus sinensis* Wiegmann) (250–253), as well as domesticated species (254–256). Farmed mink has been shown both to harbor SARS-CoV-2 and to transmit the virus to humans. Infections in farms have been reported with an estimated mutation rate of 1.16^*^10^−3^ substitutions/site/year, suggesting virus circulation (including the D614G mutation) within the mink farms for some time prior to identification (257, 258). Other animals that have been identified with SARS-CoV-2 or SARS-CoV2-like virus include tigers, lions, pigs, ferrets, golden hamsters, chickens (Gallus), and rabbits (RbCoV HKU14). Of these, pigs, cats, ferrets, and primates may have increased susceptibility to SARS-CoV-2 but possibly lesser transmission potential to humans (259–261). This lower transmission potential may be due to lower amplification potential associated with the presence of virus neutralizing antibodies or conversely a higher virulence in these animals (262, 263). The Savanna Monkeys (*Chlorocebus* spp.) are an example where a high level of human interaction occurs throughout sub-Saharan Africa and the Caribbean. Savannah Monkeys carry the ACE2 receptor for SARS-CoV-2 binding, which increases the risk of bi-directional cross-species transmission and potential of viral mutations to optimize transmission and virulence (50, 264). Ecological and environmental factors, however, may lead to natural host species that carry different coronaviruses and were initially separated geographically to come in contact in the same new host. This type of host switching and dual infection can lead to a novel recombinant virus with higher transmissibility and virulence (265).

Possible ectoparasite vectors including ticks, fleas, and mites have also been reported as transmission factors (234, 260, 266–271). However, for effective transmission and virulence, sufficient adaptation and amplification within the new host of the viral variant are required regardless of the transmission vector (272).

## Conclusion

Although direct evidence of zoonotic transfer of SARS-CoV-2 has not been demonstrated, zoonotic transfer to humans remains an ongoing concern. Recent increases in infections and the emergence of new zoonotic transmissions associated with climate change, habitat changes due to agriculture, and human-animal interaction increases the complexity of identifying possible zoonotic pathways (152, 273, 274). Research into receptor utilization has provided critical information on possible zoonotic transmission but at this stage has not identified a possible host or intermediate species for SARS-CoV, MERS, or SARS-CoV-2.

Diverse concepts aimed at explaining zoonotic transmission including phylogenetic similarities, and environmental effects, have highlighted the potential of zoonotic transmission. As such, animal hosts most closely related to humans harbor zoonoses of lower impact in terms of morbidity and mortality, while more distantly related hosts such as Chiroptera (bats) may carry highly virulent zoonoses but with a lower capacity for endemic establishment in human hosts (275). Similar environments, sharing immune response mechanisms, or cell receptors, increase the likelihood that a virus is preadapted to a novel host (66, 121, 276). The plasticity of CoVs, especially β-CoVs, allows these viruses to adapt to diverse host species en route to human spillover (277). This has been shown to be achieved through stepwise adaptive evolution of different functional proteins of SARS-CoVs at different epidemic stages and in different hosts (263, 267, 278–284). However, evidence of zoonotic transmission of specific animal CoVs has not been proven for many of the animals studied, although there is evidence that SARS-CoV may be transmitted from domestic hosts (285, 286).

Co-habitation of hosts carrying different (+)ssRNA viruses within the Coronaviridae will continue to lead to the emergence of new virulent strains by mutation, recombination, or reassortment. HCoV-NL63, an α-CoV, which leads to common cold symptoms, may be essential to follow as, in contrast to other α-CoVs, which rely on the CD13 (aminopeptidase) receptor for entry into host cells, it has been shown to lead to frequent infection in humans and utilizes the ACE2 receptor, and so may only require minor alterations to become more virulent (227, 287, 288). S-protein from HCoV-NL63 and respiratory syndrome coronavirus bind overlapping regions in ACE2. Although the factors responsible for the difference in response between SARS-CoV-2 and HCoV-NL63 are not known, several possibilities have been suggested including accessory protein incorporation into HCoV-NL63 or differences in interaction with the ACE2 receptor. The HCoV-NL63 S protein may only require minor modification to become more virulent, and this is possible due to the frequent HCoV-NL63 infections in humans and the high mutation rates in these viruses (287).

The potential for further β-CoV transmission has been highlighted by the discovery of replication-competent viruses such as bat SL-Cov-WIV1 and SL-Cov-WIV16. SL-Cov-WIV1 discovered between 2012 and 2013 has shown low-level replication in human alveolar basal epithelial (A549) cells and other mammalian cell lines. SL-Cov-WIV1 can also use ACE2 from humans and other species for cell entry (224, 226, 289).

Future mutations or recombination events of SL-CoV-WIV1 could increase virulence and the likelihood of spillover of this viral strain as a related SL-CoV-WIV16 viral mutation has already been identified with greater nucleotide similarity with SARS-CoV GZ0 human isolate from 2003 SARS outbreak. Similarly, HKU4-CoV and NL140422-CoV are capable of binding to human DPP4 without adaptation of the spike protein and pose a zoonotic risk due to possible accumulation of adaptive mutations with persistent infections (290–293). Another SARS-like virus, SHC014-CoV carrying the SHC014 spike mutation, and also found in Chinese horseshoe bats, has the potential to enter *via* ACE2 and, importantly, showed that immune-therapeutic and prophylactic modalities failed to stop the infection in a mouse model (294). The Hong Kong University 9 (HKU9) virus also has a close similarity with sections of the SARS-CoV and the Wuhan SARS-CoV-2 within the receptor-binding domain for ACE2 and is also widely distributed in diverse species including *Rousettus leschenaultii* Desmarest, *Hypostomus commersonii* Valenciennes, *Eidolon helvum* Kerr, and *R. aegyptiacus*, from Asia to Africa (20, 292, 295). Whether diversity of human ACE2 increases the potential for new zoonotic transmission of viruses with similarity to SARS-CoV and SARS-CoV-2, or as yet unidentified ACE2-binding viruses, needs to be further investigated.

The factors determining the potential for spillover have been more generally investigated from different perspectives, including phylogenetic analysis and ecogeographical perspectives. These two concepts are intricately linked as viral diversity tends to increase as ecogeographical factors become more favorable for virus to host transmission. Hence, the proportion of potentially zoonotic viruses per species can be predicted by phylogenetic relatedness to humans, host taxonomy, and extent of human population within a species range. Associated with this is the concept that ecosystems that have a large biodiversity of animals, tend to have a greater diversity of viruses. Ecogeographical factors such as clearing of land allows diverse animals that are usually isolated to interact. This is highlighted by Olival et al. (42), who reported that of their mammalian virus dataset consisting of 2,805 mammal–virus associations, and 586 viruses listed, 44.9% were found in humans, and 71.5% of human viruses had a zoonotic origin associated with virus diversity, phylogenetic proximity to humans, and opportunity for human contact (42). The viral richness or diversity in animals including bats and other mammals is most pronounced in Flaviviruses, Bunyaviruses, and Rhabdoviruses in bats (42). Marburg virus found in *R. aegyptiacus* bats and Lassa virus (LASV) found in the Natal multimammate mouse, *Mastomys natalensis* Smith (1834), across parts of Africa, further illustrate the role of geoecology of these mammals and the role this plays in the risk of zoonosis (296, 297). A similar database and extraction protocol to that discussed by Olival et al. (42) has also been developed for MERS-CoV virus host species distribution and risk of zoonotic infection, which can be used for future mapping efforts for MERS-CoV and other infectious diseases (298). In the case of a more species-restricted virus, the Epstein-Barr virus (EBV), found in *Hypsignathus monstrosus* Allen, *Eucalyptus torquate* Luehm, and *Mecynorhina torquata* Drury, fruit bats of Pteropodidae family and mainly located in Africa, modeling of possible zoonotic transmission has identified regional spread in Africa and India as a possible future outbreak risk due to bat distribution and bat-human contact probabilities (299, 300). The example of the frequent human infection associated with HCoV-NL63 suggests that highly pathogenic variants have ample opportunity to evolve different strategies for binding to the ACE2 receptor or other pathways of zoonoses (287). MERS-CoV, but not HKU4, has adapted to use human DPP4 and human cellular proteases for efficient human cell entry, contributing to the enhanced pathogenesis of MERS-CoV in humans. A similar risk of future zoonosis is associated with the capability of the S protein to adapt to humans for cross-species transmissions, as may be the case with the HKU4 virus, which binds to DPP4 receptors but with current affinity to bat DPP4 rather than the human DPP4 (291). To understand the zoonotic potential of coronaviruses, further research is required into the role of the ORF genes as well as viral non-structural proteins responsible for the replication and transcription of the viral genome (15). ACE2 receptor variants may also contribute to identifying potential future zoonotic transmission and spillover to other mammals (301).

Determining zoonotic niches for viral host species and continued surveillance of cases of viral infection also play an essential role in predicting the risk and potential spread of zoonotic transmission with reference to virus evolution and ecology. Reverse zoonoses followed by secondary infection may play an important part in SARS-CoV-2 viral transmission and virulence (8, 302). Human to animal host leads to further mutations of the virus in humans which may, in some cases, lead to an improved adaptability of the virus and hence enhanced spread and infection back to humans. Genetic sequence homology between coronaviruses including MERS and SARS-CoV2 may lead to combined infection in human hosts as a function of dual transmission, especially in North Africa and in the Middle East and North Africa (MENA) region in general. Dual infection can then lead to viral mutations in human hosts that may spillover back to animals and be more virulent than the original. This possibility needs to be closely monitored (265). Viral mutations in spillover species such as mink and domesticated animals also needs to be monitored as SARS-CoV-2 mutations in some animal species have already been observed that may change the virulence of SARS-CoV-2 and reduce the likelihood of SARS-CoV-2 losing either its virulence and/or transmissibility. Future pandemics may not only come from α-CoV or β-CoV but also from γ-CoV that are found in domestic birds such as turkeys, guinea fowls, or quails and more recently in beluga whales and bottleneck dolphins (303–305). δ-CoVs have potential to spillover to humans and are present in different mammalian (Asian leopard cat CoV, Chinese ferret badger CoV, porcine CoV HKU15) and avian (bulbul CoV HKU11, thrush CoV HKU12, munia CoV HKU13, white-eye CoV HKU16, sparrow CoV HKU17, magpie-robin CoV HKU18, night heron CoV HKU19, wigeon CoV HKU20, and common moorhen CoV HKU21) species (159, 306–308).

## Author Contributions

HJ, MM, EA, WO, IS, DB, GT, and HA contributed to conception and design of the study. HJ took the lead in writing the manuscript. A comprehensive search using Ovid software on Global Health, Medline, PubMed, Zoological Record was performed by MM. GT and HA supervised the project. All authors contributed to manuscript revision, read, and approved the submitted version.

## Conflict of Interest

The authors declare that the research was conducted in the absence of any commercial or financial relationships that could be construed as a potential conflict of interest. The reviewer AV declared a shared affiliation with one of the authors JB, to the handling Editor at time of review.
