# Corrigendum: Evolution, ecology, and zoonotic transmission of betacoronaviruses: A review

**DOI:** 10.3389/fvets.2023.1147940

**Published:** 2023-02-08

**Authors:** Herbert F. Jelinek, Mira Mousa, Eman Alefishat, Wael Osman, Ian Spence, Dengpan Bu, Samuel F. Feng, Jason Byrd, Paola A. Magni, Shafi Sahibzada, Guan K. Tay, Habiba S. Alsafar

**Affiliations:** ^1^Center for Biotechnology, Khalifa University of Science and Technology, Abu Dhabi, United Arab Emirates; ^2^Department of Biomedical Engineering, College of Engineering, Khalifa University of Science and Technology, Abu Dhabi, United Arab Emirates; ^3^Center of Heath Engineering Innovation, Khalifa University of Science and Technology, Abu Dhabi, United Arab Emirates; ^4^Nuffield Department of Women's and Reproduction Health, Oxford University, Oxford, United Kingdom; ^5^Department of Pharmacology, College of Medicine and Health Sciences, Khalifa University of Science and Technology, Abu Dhabi, United Arab Emirates; ^6^Department of Biopharmaceutics and Clinical Pharmacy, School of Pharmacy, The University of Jordan, Amman, Jordan; ^7^Department of Chemistry, College of Arts and Sciences, Khalifa University of Science and Technology, Abu Dhabi, United Arab Emirates; ^8^Discipline of Pharmacology, University of Sydney, Sydney, NSW, Australia; ^9^State Key Laboratory of Animal Nutrition, Institute of Animal Science, Chinese Academy of Agricultural Science, Beijing, China; ^10^Department of Mathematics, Khalifa University of Science and Technology, Abu Dhabi, United Arab Emirates; ^11^Department of Pathology, Immunology and Laboratory Medicine, University of Florida, Gainesville, FL, United States; ^12^Discipline of Medical, Molecular and Forensic Sciences, Murdoch University, Murdoch, WA, Australia; ^13^Murdoch University Singapore, King's Centre, Singapore, Singapore; ^14^Antimicrobial Resistance and Infectious Diseases Laboratory, College of Science, Health, Engineering and Education, Murdoch University, Murdoch, WA, Australia; ^15^Division of Psychiatry, Faculty of Health and Medical Sciences, The University of Western Australia, Crawley, WA, Australia; ^16^School of Medical and Health Sciences, Edith Cowan University, Joondalup, WA, Australia; ^17^Department of Genetics and Molecular Biology, College of Medicine and Health Sciences, Khalifa University of Science and Technology, Abu Dhabi, United Arab Emirates

**Keywords:** zoonoses, coronavirus, SARS-CoV-2, zoonotic transmission, ecology, evolution, reservoir species

In the published article, **Plowright et al. (2017)** was not cited in the article. The citation has now been inserted in **the Introduction**, **First Paragraph** and the updated reference list appears below.

“The emergence of the Severe Acute Respiratory Syndrome Coronavirus 2 (SARS-CoV-2), the virus responsible for the COVID-19 pandemic, has focused attention on the phenomenon of zoonosis. Zoonoses, as defined by the World Health Organization (WHO), are diseases and infections which are naturally transmitted between vertebrate animals and humans. The challenge with emerging zoonoses, such as SARS-CoV-2, is to establish the origin and mechanism(s) of transmission of the new disease. Viral mutation and recombination, viral host physiology and immune response, ecogeography, and human factors including ACE2 receptor structure and immune function have all been proposed as possible mechanisms (1, 2) (Figure 1).”

The authors apologize for this error and state that this does not change the scientific conclusions of the article in any way. The original article has been updated.
