# Corrigendum: Evolution, ecology, and zoonotic transmission of betacoronaviruses: a review

**DOI:** 10.3389/fvets.2023.1215156

**Published:** 2023-05-17

**Authors:** Herbert F. Jelinek, Mira Mousa, Eman Alefishat, Wael Osman, Ian Spence, Dengpan Bu, Samuel F. Feng, Jason Byrd, Paola A. Magni, Shafi Sahibzada, Guan K. Tay, Habiba S. Alsafar

**Affiliations:** ^1^Center for Biotechnology, Khalifa University of Science and Technology, Abu Dhabi, United Arab Emirates; ^2^Department of Biomedical Engineering, College of Engineering, Khalifa University of Science and Technology, Abu Dhabi, United Arab Emirates; ^3^Center of Heath Engineering Innovation, Khalifa University of Science and Technology, Abu Dhabi, United Arab Emirates; ^4^Nuffield Department of Women's and Reproduction Health, Oxford University, Oxford, United Kingdom; ^5^Department of Pharmacology, College of Medicine and Health Sciences, Khalifa University of Science and Technology, Abu Dhabi, United Arab Emirates; ^6^Department of Biopharmaceutics and Clinical Pharmacy, School of Pharmacy, The University of Jordan, Amman, Jordan; ^7^Department of Chemistry, College of Arts and Sciences, Khalifa University of Science and Technology, Abu Dhabi, United Arab Emirates; ^8^Discipline of Pharmacology, University of Sydney, Sydney, NSW, Australia; ^9^State Key Laboratory of Animal Nutrition, Institute of Animal Science, Chinese Academy of Agricultural Science, Beijing, China; ^10^Department of Mathematics, Khalifa University of Science and Technology, Abu Dhabi, United Arab Emirates; ^11^Department of Pathology, Immunology and Laboratory Medicine, University of Florida, Gainesville, FL, United States; ^12^Discipline of Medical, Molecular and Forensic Sciences, Murdoch University, Murdoch, WA, Australia; ^13^Murdoch University Singapore, King's Centre, Singapore, Singapore; ^14^Antimicrobial Resistance and Infectious Diseases Laboratory, College of Science, Health, Engineering and Education, Murdoch University, Murdoch, WA, Australia; ^15^Division of Psychiatry, Faculty of Health and Medical Sciences, The University of Western Australia, Crawley, WA, Australia; ^16^School of Medical and Health Sciences, Edith Cowan University, Joondalup, WA, Australia; ^17^Department of Genetics and Molecular Biology, College of Medicine and Health Sciences, Khalifa University of Science and Technology, Abu Dhabi, United Arab Emirates

**Keywords:** zoonoses, coronavirus, SARS-CoV-2, zoonotic transmission, ecology, evolution, reservoir species

In the published article, there was an error in the legend for Figure 1 as published.

The figure legend did not indicate that it has been adapted from Plowright et al. (2017). Copyright permission was obtained from Springer Nature to adapt Figure 1 from Plowright et al. (2017).

The corrected legend appears below.

Figure 1. Zoonotic risk distribution, pathway to spillover, and the multimodal role of the determinants of spillover. The zoonotic risk is demonstrated by the accumulated distribution of reservoir hosts and vectors that play a role in the pathway to spillover. The risk of spillover is determined by a series of processes from the ecological dynamics of reservoir host distribution and density, to the susceptibility, replication and dissemination of the biological factors in the recipient host. This is also reflected in the multimodal role of the determinants of spillover, demonstrating the disciplines that are being used to study zoonotic transmission and the determinants of spillover. This figure was adapted from Figure 1 in Plowright et al. (2). Copyright permission was obtained with license number 55218980848529.

The authors apologize for this error and state that this does not change the scientific conclusions of the article in any way. The original article has been updated.

